# Poisoning Caused by Medicines and Drugs of Abuse

**DOI:** 10.3390/toxics10090515

**Published:** 2022-08-31

**Authors:** Elisabetta Bertol, Claudia Trignano

**Affiliations:** 1 Research Unit U.R.I.To.N., University of Firenze, 50134 Firenze, Italy; 2 Department of Biomedical Sciences, University of Sassari, 07100 Sassari, Italy

We believe it is necessary to state a premise on the framing of poison and poisoning in the context of Forensic Toxicology as an important contribution to this Special Issue, which is composed of articles about this discipline—the discipline of “poison”.

Poison refers to any substance (solid, liquid, or gas) which, if assimilated in or brought into contact with any part of the living body, leads to the deterioration of health or, eventually, death through its constitutional or local effects. Every agent may be harmful if acting on an organism at a high enough dose. 

Forensic Toxicology was born of the need for justice in the field of poisoning (accidental, suicidal, or fraudulent), so the most important task in this discipline is analyzing biological samples for the presence of toxics, including medicines and drugs of abuse.

The field of Forensic Toxicology involves other sub-disciplines: postmortem forensic toxicology, human performance toxicology, and forensic drug testing. All these sub-disciplines measure substances in biological matrices for a given purpose. The primary concerns of forensic toxicology investigators include determining whether a harmful substance can cause death, impair judgment (e.g., when driving or in the workplace), and change behavior, or whether it has a legitimate presence in the body.

Drug toxicity can result from the over-ingestion of medicines, causing too much of the drug to be in a person’s system at once. This can happen if the dose taken exceeds the prescribed amount, or if the prescribed dosage is too high. For drugs of abuse, most of which are psychoactive drugs leading to tolerance, the poisoning happens when the dose exceeds the individual’s tolerance.

This Special Issue aims to highlight and collect research on the established knowledge, as well as open issues, related to drug poisoning; it aims to depict the state of the art and provide new starting points for future advances, especially for new substances (NPS) and their mechanisms of action, while not forgetting the problems of traditional drugs, which are often the most involved in mortality.

We collected eleven contributions (articles, reviews, and case reports).

Regarding the articles, the predictors of mortality in illicit-drug users involving Novel Psychoactive Substances (NPS) are defined in the NPS endemic era in the paper “Clinical Presentations and Predictors of In-Hospital Mortality in Illicit Drug Users in the New Psychoactive Substances (NPS) Endemic Era in Taiwan” by a team of researchers at the University of Taiwan [1].

In an interesting article by the Department of Pharmacology and Toxicology (College of Pharmacy, University of Saudi Arabia), “Effects of Chronic Inhalation of Electronic Cigarette Vapor Containing Nicotine on Neurobehaviors and Pre/Postsynaptic Neuron Markers”, the researchers present an experimental study concerning nicotine-exposed animal models that exhibit neurobehavioral changes linked to impaired synaptic plasticity. Such exposure was also associated with altered neurobehaviors, which might affect neurodegenerative diseases [2].

“Suicidal Behavior and Its Relationship with Postmortem Forensic Toxicological Findings” is presented by a group of authors at Murcia University and the Murcia Pathology Service (Institute of Legal Medicine and Forensic Sciences). They emphasize that toxicological analysis is fundamental to understanding consumption patterns and in establishing strategies and protocols for detecting and preventing suicide [3].

In their paper “A Forensic Diagnostic Algorithm for Drug-Related Deaths: A Case Series”, an Italian team presents a diagnostic algorithm for coroners with all the elements for investigating drug-related deaths and cooperating with toxicologists. Toxicological forensic diagnosis is still extremely heterogeneous in regional and national contexts. Funding for improvements to method development, research, networking, facilities, and technologies is mandatory to standardize toxicological investigations [4].

A paper titled “The Clinical Presentations of Nitrous Oxide Users in an Emergency Department of a Taiwan Hospital”, in collaboration with the university, illustrates that patients who present a more severe triage level, a faster heart rate, greater agitation, and cardiovascular symptoms were significantly noted to be combined-illicit-drug users. The authors conclude that the combined use of N_2_O and illicit drugs can cause great harm [5].

The effects of the prolonged use of Cannabis are discussed in an Italian paper titled “Neuronal and Astrocytic Morphological Alterations Driven by Prolonged Exposure with Δ9-Tetrahydrocannabinol but Not Cannabidiol”. The results suggest that prolonged exposure to THC or CBD induces different effects in the hippocampus. In particular, 72 h of THC exposure induced neuronal and glial alterations that must draw our attention to the possible effects relatively prolonged use, especially in adolescents [6].

Some reviews are also present in this Special Issue. One of these—“Is Illicit Substance Use Gender-Specific? The Basic Points of Mental and Health Disorders”, by authors at the University of Zagora, Bulgaria—purposes the provision of general data on the relationship between different psychostimulants, clinical and socio-demographic studies, and gender, both among the general population and in one of the most at-risk groups. The study may provide some guidance for developing gender-specific treatment and prevention, including responses to some pharmacological and behavioral therapies. The review is intended for a wide audience of social workers, toxicologists, and pharmacologists [7].

Another review from the same authors at the University of Zagora, Bulgaria—“Oxidative Stress and Cocaine Intoxication as Start Points in the Pathology of Cocaine-Induced Cardiotoxicity”—concerns cocaine as a classic stimulant, and its cardiotoxicity. The aim of the review is to assess acute and chronic cocaine toxicity by focusing on the published literature regarding oxidative stress levels. Hypothetically, this study can serve as a basis for developing a rapid and effective method for determining oxidative stress levels by monitoring changes in the redox status of patients with cocaine intoxication [8].

Pharmacogenetics is addressed in the paper “Pharmacogenetics and Forensic Toxicology: A New Step towards a Multidisciplinary Approach” by Italian researchers at University of Lecce (Foggia, Catania and Palermo). The aim of this systematic literature review is not only to raise awareness among the forensic community about pharmacogenetics, but also to provide a workflow for forensic toxicologists in cases of unknown causes of death related to drug use/abuse. This study highlights the importance of forensic pharmacogenetics, a field of toxicology that is still not fully understood; it is of great help in cases of sudden death, deaths from overdose, deaths after the administration of a drug, and also in cases of medical malpractice complaints [9].

Case reports are also part of this Special Issue, such as the paper “Peroxisome Proliferator-Activated Receptor Delta Agonist (PPAR-δ) and Selective Androgen Receptor Modulator (SARM) Abuse: Clinical, Analytical and Biological Data in a Case Involving a Poisonous Combination of GW1516 (Cardarine) and MK2866 (Ostarine)” by French researchers (Legal Medicine of Strasbourg, the Centre Antipoison et de Toxicovigilance of Bordeaux, and the Service des Urgences of Marmande). The paper concerns a 43-year-old male sports coach, who presented at the emergency unit of a local hospital for epigastric pain, myalgia pain, and severe headache after having used a combination of GW1516 (cardarine) and MK2866 (ostarine) for several days to gain skeletal muscle. Both drugs were identified in 2 cm of the patient’s hair, demonstrating repetitive abuse over the last 2 months [10].

Another case report is illustrated in this article—“Overdose of Quetiapine—A Case Report with QT Prolongation”, by Italian Authors at the Florence, Padua, Sassari, and Palermo Universities—involving a rare case of overdose of this substance, although several studies describe the adverse effects of intoxication with Quetiapine, an atypical antipsychotic drug. The case concerns a 75-year-old male who attempted suicide by ingesting 28 g of Quetiapine. During management in the emergency department, both serum and urine samples were collected, allowing a complete pharmacokinetic analysis to be conducted from admission to discharge [11].

The original articles, reviews, and case reports in this Special Issue represent the various aspects of poisoning and forensic toxicology topics that we set out to address and include suggestions for treating some cases of intoxication. For some uncommon substances they also represent an acquisition of new knowledge that may be useful in the discipline.
